# Entropy and Chaos-Based Modeling of Nonlinear Dependencies in Commodity Markets

**DOI:** 10.3390/e27090955

**Published:** 2025-09-14

**Authors:** Irina Georgescu, Jani Kinnunen

**Affiliations:** 1 Department of Economic Informatics and Cybernetics, Bucharest University of Economics, Calea Dorobanți, 010552 Bucharest, Romania; 2 School of Business, LUT University, Yliopistonkatu 34, 53851 Lappeenranta, Finland; jani.kinnunen@abo.fi

**Keywords:** chaos theory, Shannon entropy, mutual information, wavelet coherence, SO(2) lie group, commodity markets

## Abstract

This study explores the nonlinear dynamics and interdependencies among major commodity markets—Gold, Oil, Natural Gas, and Silver—by employing advanced chaos theory and information-theoretic tools. Using daily data from 2020 to 2024, we estimate key complexity measures including Lyapunov exponents, correlation dimension, Shannon and Rényi entropy, and mutual information. We also apply the stochastic SO(2) Lie group method to model dynamic correlations, and wavelet coherence analysis to detect time-frequency co-movements. Our findings reveal evidence of low-dimensional deterministic chaos and time-varying nonlinear relationships, especially among pairs like Gold–Silver and Oil–Gas. These results highlight the importance of using nontraditional approaches to uncover hidden structure and co-movement dynamics in commodity markets, providing useful insights for portfolio diversification and systemic risk assessment.

## 1. Introduction

Understanding the dynamic relationships among key financial and commodity markets has become increasingly important in the context of heightened global uncertainty, volatility, and structural transformations. Traditional econometric models, while useful for capturing linear trends and mean-reverting behavior, often fail to detect the presence of chaotic patterns, nonlinear dependencies, and time-varying co-movements that are critical for forecasting, hedging, and diversification strategies.

Recent advances in nonlinear time series analysis, complexity theory, and geometric modeling have opened new avenues for examining the hidden dynamics underlying asset behavior. In particular, tools such as Lyapunov exponents and correlation dimension provide quantitative diagnostics for chaos, while entropy-based measures such as Shannon and Rényi entropy offer insights into unpredictability and information content. Mutual information, unlike standard correlation, captures both linear and nonlinear dependencies, making it particularly well suited for studying asset interrelationships in turbulent market conditions.

The incorporation of geometric and topological frameworks—such as Lie group SO(2) rotations and wavelet coherence—allows for the construction of time-varying correlation models that respect the underlying mathematical structure of the data. These approaches have been successfully applied in recent literature to explore complex interactions in markets as diverse as cryptocurrency, interest rates, and energy commodities.

While significant progress has been made in modeling financial and commodity markets using tools from nonlinear dynamics, chaos theory, and information geometry, existing studies often focus narrowly on either isolated chaotic behavior or partial dependence structures. Traditional econometric and GARCH-based approaches remain dominant but are limited in capturing the full spectrum of nonlinearities and evolving interdependencies. Although some recent works have incorporated Lie group structures or entropy measures, very few integrate a comprehensive framework that combines chaos diagnostics (e.g., Lyapunov exponents, correlation dimension), entropy-based complexity, nonlinear dependence via mutual information, and geometrically consistent dynamic correlations modeled through SO(2) Lie group rotations. Wavelet coherence remains underutilized in synergy with these methods for analyzing time–frequency co-movements. This study addresses the gap by unifying these methodologies to explore the hidden dynamics and interdependencies of major commodities, offering a novel approach to systemic risk and portfolio management.

Building on these developments, this paper provides a comprehensive framework for analyzing the nonlinear and time–frequency characteristics of major commodity markets. By combining empirical chaos diagnostics, information-theoretic dependence measures, geometric correlation models, and wavelet coherence analysis, we offer a robust methodology for understanding how financial assets interact over time and across different time scales. All computations and empirical analyses were conducted using RStudio (version 2023.12.1+402).

## 2. Literature Review

Modeling time-varying and nonlinear dependencies in financial and commodity markets has gained increasing attention in recent literature, particularly in the context of systemic risk, portfolio diversification, and dynamic hedging under market turbulence. Traditional approaches—such as rolling-window correlations and multivariate GARCH models—often fail to account for the geometric properties of correlation matrices or the underlying nonlinear and chaotic dynamics in asset returns. In response, researchers have developed alternative methodologies rooted in complexity theory, information geometry, and Lie group structures.

One notable advancement is the use of Lie group theory to construct dynamically consistent correlation matrices. Muniz et al. (2021) [1] introduced a stochastic differential equation framework constrained to the special orthogonal group SO(n), ensuring that time-varying correlation matrices remain valid (i.e., symmetric, positive semi-definite, and orthogonal). Their use of isospectral flows and Lie group integrators represents a robust way to simulate geometrically consistent correlation dynamics.

This line of research has been extended by Bildirici et al. (2025) [2], who propose a stochastic SO(2) Lie group rotation model to capture the dynamic interaction between financial assets. By introducing stochasticity via Brownian motion into the rotation angle, their approach preserves the structural integrity of correlation matrices while also modeling the nonlinearity and irregularity commonly observed in commodity prices. Their empirical findings highlight the effectiveness of this method in capturing hidden co-movement patterns, especially during episodes of volatility clustering and regime switching.

In parallel, the application of chaos theory has expanded in empirical finance. Recent studies have employed Lyapunov exponents, correlation dimension, and nonlinear entropy measures to detect deterministic chaos and complexity in financial time series. Atik et al. (2024) [3] examine the nonlinear tail dependence and risk spillovers between large cryptocurrencies, stablecoins, and commodity markets before and after major monetary policy shifts, documenting persistent bi-directional tail co-movements that conventional models fail to capture. Papla and Siedlecki (2024) [4] investigate entropy dynamics during major economic transitions, focusing on three distinct periods: the pre-pandemic phase (2015–2019), the COVID-19 pandemic (2020–2021), and the geopolitical shock following Russia’s invasion of Ukraine (2022–2023). The analysis is based on daily price data for electricity, oil, coal, and gas across 27 European Union countries and Norway, spanning from 1 January 2015 to 30 March 2023. By employing two-dimensional configurations of electricity and commodity prices, the study applies a time-varying James–Stein estimator of Shannon entropy to capture fluctuations in informational complexity across different market regimes. Drzazga-Szczęśniak et al. (2023) [5] analyze the entropy of the Polish stock market before and after the outbreak of the 2022 Russia–Ukraine war. Their findings show that entropy effectively detects shifts in market volatility induced by extreme external events, outperforming traditional measures like standard deviation. Georgescu et al. (2024) [6] investigate the main drivers of electricity prices in Romania’s day-ahead market between 2019 and 2022, a period marked by major disruptions such as the COVID-19 pandemic and the war in Ukraine. Using advanced techniques like Principal Component Analysis (PCA), Continuous Wavelet Transform (CWT), and the Herfindahl-Hirschman Index (HHI), the authors analyze the interplay between market competitiveness, energy fundamentals, and macroeconomic conditions.

The measurement of nonlinear dependence using mutual information (MI) has also gained prominence. While Pearson correlation remains widely used, MI can detect both linear and nonlinear associations. Kraskov et al. (2004) [7] and more recently, Chvosteková et al. (2021) [8] proposed data-efficient MI estimators that outperform standard methods in detecting cross-asset dependencies in volatile markets. MI has also been successfully integrated into network analysis, revealing hidden clusters and hubs in financial systems under stress by Chen et al. (2022) [9].

Another stream of research applies wavelet coherence to financial time series to study co-movements across both time and frequency domains. Aguiar-Conraria and Soares (2011) [10] laid the groundwork for this method in economics. A more recent study by Szczygielski et al. (2023) [11] applies wavelet coherence to examine uncertainty spillovers from stock markets—proxied by both the VIX and Google search trends—into various commodity markets. Their analysis reveals that energy commodities are especially vulnerable to such spillovers, with coherence levels peaking at multiple time horizons during significant events such as the COVID-19 crisis, underscoring the time-frequency dimension of risk transmission.

A growing body of research explores innovative mathematical tools for modeling economic and financial dynamics. For instance, Makowski and Piotrowski (2024) [12] employ the Radon transform to develop a non-stochastic, geometric model of financial risk, offering an alternative to volatility-based approaches. In parallel, fractional calculus has been increasingly adopted in macroeconomic and financial modeling. Cheow et al. (2024) [13] apply fractional calculus to economic growth modeling, showing that fractional-order derivatives can effectively capture memory effects and long-range dependence in GDP dynamics. Their results prove that fractional models provide a more flexible and accurate representation of growth processes compared to traditional integer-order approaches. Johansyah et al. (2021) [14] provide a systematic review of the use of fractional differential equations in economic growth models, with a focus on the role of memory effects and the development of solution techniques. Their study highlights both linear and nonlinear formulations, including applications of the fractional Riccati differential equation, and identifies gaps for future research in modeling economic processes with long-term dependence. Tejado and Valério (2019) [15] develop fractional-order differential equation models of GDP growth for the G7 economies, comparing them against integer-order models. Their results show that fractional models achieve superior fit and short-term predictive accuracy without increasing the number of parameters. Kacapor et al. (2025) [16] apply Grünwald–Letnikov fractional-order calculus to model Serbia’s GDP growth, comparing it against a standard integer-order model. Their results show that the fractional model provides a substantially better fit and more accurate predictions. All these examples confirm a broader trend of advanced mathematical techniques applied to capture nonlocality, memory, and complex dependencies.

These contributions highlight the increasing role of geometry-aware, chaos-sensitive, and frequency-decomposing methods in financial econometrics. They provide a comprehensive framework for understanding asset return dynamics beyond linearity and stationarity, thus justifying the integrated methodological approach adopted in this study.

The paper is structured as follows. Section 1 introduces the motivation for analyzing commodity markets through the lens of nonlinear dynamics, entropy, and geometric modeling. Section 2 provides a review of the relevant literature, highlighting recent advances in chaos theory, entropy-based complexity, wavelet coherence, and Lie group methods. Section 3 outlines the methodological framework, including the computation of Lyapunov exponents, correlation dimension, Shannon and Rényi entropy, mutual information, SO(2) Lie group-based correlation modeling, and wavelet coherence analysis. Section 4 reports the empirical results across all methodological layers, offering insights into dynamic interdependencies, nonlinear structures, and time–frequency behavior. Section 5 concludes and offers suggestions for future research directions.

## 3. Methodological Framework

**Figure 1:**
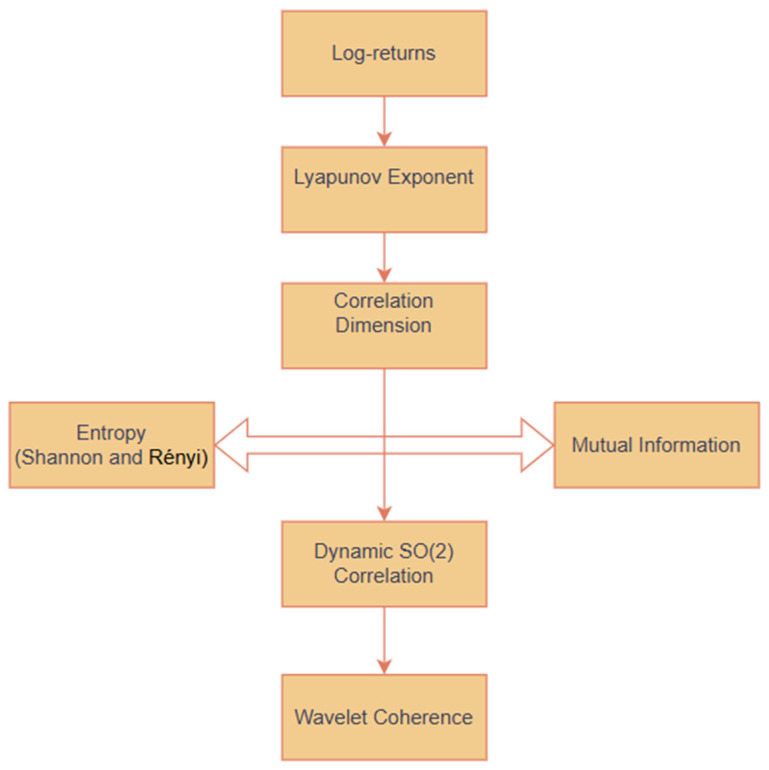
Methodological flow.

Figure 1 outlines the study’s methodological framework, combining chaos theory, entropy, mutual information, SO(2) dynamic correlations, and wavelet coherence.

### 3.1. Lyapunov Exponent

The Lyapunov exponent is a central concept in the study of nonlinear dynamical systems and chaos theory, used to quantify the rate at which nearby trajectories in phase space diverge. It characterizes the sensitivity of a system to initial conditions, and its positivity is a strong indicator of deterministic chaos (Strogatz, 2018 [17]; Ott, 2002 [18]). In applied contexts such as finance and physics, it serves as a diagnostic measure to differentiate between random fluctuations and underlying chaotic structure. In financial time series analysis, a positive Lyapunov exponent has been interpreted as evidence of deterministic, yet unpredictable dynamics, especially in contexts such as asset prices, interest rates, and exchange rate volatility (Peters, 1994 [19]; Scheinkman & LeBaron, 1989 [20]).

Let a deterministic system evolve according to a smooth mapping $f:R^{n}\to R^{n}$, generating the sequence:

(1) $$x_{n+1}=f\left(x_{n}\right)$$

Let $\delta x_{0}$ be a small initial perturbation. Then, the evolution of this deviation over time follows:

(2) $$∥\delta x_{t}∥\approx ⟦\delta x_{0}⟧e^{\lambda t}$$

where λ is the Lyapunov exponent. If λ>0, the separation between trajectories grows exponentially, leading to unpredictability in log-term forecasting even when the system is entirely deterministic. Further methodological and computational details are provided in Appendix A.1.

### 3.2. Correlation Dimension (Grassberger–Procaccia Method)

D_2_ is a fundamental nonlinear invariant used to characterize the complexity or fractal nature of a dynamical system’s attractor. It provides a quantitative measure of how densely the points of a reconstructed trajectory occupy the phase space and is particularly effective in distinguishing chaotic from stochastic signals (Grassberger & Procaccia, 1983 [21]).

The Grassberger–Procaccia algorithm begins by reconstructing the attractor from a scalar time series using Takens’ embedding theorem. This involves creating delay vectors in an m-dimensional space as in Equation (A2).

Once the phase space is reconstructed, the correlation sum C(r) is computed:

(3) $$C\left(r\right)=\underset{N\to \infty }{\lim}\frac{2}{N\left(N-1\right)}\overset{N}{\underset{i=1}{\sum }}\overset{N}{\underset{j=i+1}{\sum }}H(r-∥x_{i}-x_{j}∥)$$

In Equation (3). N is the number of delay vectors, ∥.∥ denotes the Euclidean norm, and H(.) is the Heaviside step function, counting the number of pairs of points within a radius r.

For a fractal attractor, the correlation sum scales with the radius r as:

(4) $$C\left(r\right)\propto r^{D_{2}}$$

Hence, the correlation dimension $D_{2}$ is estimated from the slope of the log-log plot:

(5) $$D_{2}=\underset{r\to \infty }{\lim}\frac{d\log C\left(r\right)}{d\log\;r}$$

In practice, one searches for a linear region in the log C(r) versus log r plot over an appropriate scaling range. The slope of this region yields the correlation dimension, which serves as a measure of system complexity. Further methodological details, including algorithmic steps and implementation in R, are provided in Appendix A.2.

### 3.3. Entropy Measures: Shannon and Rényi Entropy

To assess the complexity and uncertainty inherent in the financial time series, we employed entropy-based measures—specifically, Shannon entropy and Rényi entropy—which quantify the unpredictability of a system’s dynamics. These measures provide complementary insights into the degree of randomness and distributional characteristics of asset returns.

Shannon entropy, introduced by Claude Shannon (1948) [22], is a foundational concept in information theory. It is defined for a discrete probability distribution $\left\{p_{i}\right\}$ as:

(6) $$H=-\overset{n}{\underset{i=1}{\sum }}p_{i\;}\log p_{i}$$

where $p_{i\;}$ denotes the probability of observing a value within the i-th histogram bin. In our implementation, we estimated the empirical probability density function of the asset log-returns using fixed-width bin histograms and applied the formula to evaluate the entropy of each return series. Higher values of Shannon entropy indicate greater unpredictability and less information redundancy in the time series (Shannon, 1948 [22]).

Although Shannon entropy is originally defined for discrete probability distributions, our data consist of continuous-valued financial returns. To bridge this gap, we discretized the return distributions into histogram bins and then applied the Shannon entropy formula to the empirical probability mass function. This discretization-based approach is widely used in nonlinear time series and econophysics to approximate entropy in continuous systems (see Cover & Thomas, 2006 [23]). An alternative would be the use of differential entropy, the continuous analog of Shannon entropy, which, however, has interpretability challenges (e.g., it can take negative values and depends on the choice of units). The discretized Shannon entropy used here provides a robust and comparable measure of unpredictability across assets, consistent with established practice in financial time series analysis (e.g., Papla and Siedlecki, 2025 [4]).

In addition to Shannon’s measure, we used Rényi entropy, which generalizes the Shannon formulation and introduces a parameter α > 0 that controls the sensitivity to distribution tails. The Rényi entropy of order α is given by:

(7) $$H_{\alpha }=\frac{1}{1-\alpha }\log(\overset{n}{\underset{i=1}{\sum }}p_{i}^{\alpha })$$

When α = 2, the Rényi entropy emphasizes more probable outcomes and is particularly useful for assessing heavy-tailed distributions often present in financial data (Rényi, 1961 [24]). We used α = 2 (quadratic Rényi entropy), which is closely connected to the correlation dimension. This order is commonly employed in nonlinear dynamics and chaos analysis, as it emphasizes dominant patterns while retaining sensitivity to heavy tails, making it particularly appropriate for financial return distributions (Dlask & Kukal, 2018 [25]).

The entropy measures were implemented in R using a custom function that calculates both indices based on histogram-estimated probabilities. The use of both entropy measures helps reveal subtle differences in dynamical behavior between assets, supporting a robust assessment of their chaotic properties and information content.

### 3.4. Mutual Information

To examine the dependency structure between asset pairs, we computed Mutual Information (MI)—a nonparametric, entropy-based measure of statistical dependence between two time series. Unlike Pearson correlation, which captures only linear associations, MI detects both linear and nonlinear relationships, making it particularly suitable for financial time series characterized by complexity and potential nonlinearity. For two time series X and Y, the mutual information $I\left(X;Y\right)$ is defined as:

(8) $$I\left(X;Y\right)=H\left(X\right)+H\left(Y\right)-H\left(X,Y\right)$$

where H(X) and H(Y) are the marginal Shannon entropies of the variables, and H(X,Y) is their joint entropy (Shannon, 1948 [22]). This formulation quantifies the reduction in uncertainty of one variable due to knowledge of the other. When X and Y are independent, $I\left(X;Y\right)=0$; greater values imply stronger dependencies. Further methodological and implementation details are provided in Appendix A.3.

### 3.5. Dynamic Correlations via Lie Group SO(2) Rotations

This part of the methodology is inspired by the work of Bildirici et al. (2025) [2], who applied the stochastic SO(2) Lie group framework to model the dynamic correlation between Bitcoin and gold prices. Their study proved that traditional correlation measures might fail to capture the complex, chaotic, and fractional dynamics present in financial time series. To capture the time-varying structure of financial correlations, we implement a model based on stochastic rotations of the covariance matrix using the special orthogonal group SO(2). The group SO(2) comprises all 2 × 2 real orthogonal matrices with determinant 1, representing planar rotations. This Lie group structure preserves both length and angle—making it ideal for modeling smooth, reversible deformations of a correlation system over time. Further details on the stochastic evolution of the rotation parameter and the derivation of time-varying correlation coefficients are provided in Appendix A.4.

### 3.6. Mutual Information Network Construction

To explore the structural dependencies between financial assets, we construct a mutual information network using the igraph package in R. In this network, each node represents an asset (e.g., Gold, Oil, Gas, Silver), while edges signify statistically significant MI values, specifically those exceeding a threshold of I > 0.01. The weight of each edge corresponds to the strength of mutual information, capturing both linear and nonlinear dependencies between time series, unlike traditional Pearson correlation which detects only linear relationships.

We compute betweenness centrality for each node. This metric quantifies the frequency with which a node acts as a bridge along the shortest paths between pairs of other nodes. Mathematically, the betweenness centrality $C_{B}\left(v\right)\;$for a node v is defined as:

(9) $$C_{B}\left(v\right)=\underset{s\neq v\neq t}{\sum }\frac{\sigma_{st}\left(v\right)}{\sigma_{st}}$$

where $\sigma_{st}$ is the number of shortest paths between nodes s and t, and $\sigma_{st}$ is the number of those paths that pass through node v. This measure provides an indication of the relative importance or influence of an asset in facilitating information flow across the network (Newman, 2010 [26]). In our application, the node size is scaled proportionally to its betweenness centrality, visually highlighting the most central or influential assets in terms of mutual information connectivity.

The construction of such networks allows for a richer and more nuanced understanding of market interdependencies, especially under conditions of uncertainty or financial stress, where nonlinear relationships tend to dominate. The methodological advantage of mutual information networks lies in their ability to uncover hidden channels of co-movement that would be missed by conventional correlation-based approaches (Kraskov et al., 2004 [7]).

### 3.7. Time–Frequency Dependency via Wavelet Coherence

Wavelet coherence is a powerful method that enables the examination of the localized co-movement between two time series across both time and frequency domains. Unlike traditional correlation measures, which provide a single, static value, wavelet coherence allows researchers to explore how the relationship between two series evolves over time and at different frequencies. This is particularly useful in financial and commodity markets, where asset interdependencies are often nonlinear, time-varying, and affected by structural breaks or market shocks (Torrence & Compo, 1998 [27]; Grinsted et al., 2004 [28]).

In this study, wavelet coherence complements traditional correlation and nonlinear dependence measures such as mutual information and Lyapunov exponents. It offers a nuanced and detailed view of time-varying asset interdependencies, providing valuable insights for portfolio diversification, risk management, and the identification of contagion channels in financial systems. Further methodological details, including the formal definitions of the wavelet transform and coherence, significance testing procedures, and implementation steps, are provided in Appendix A.5.

## 4. Empirical Results

This section presents the empirical results of a chaos and complexity investigation conducted on a four-asset portfolio consisting of Gold, Oil, Natural Gas, and Silver. The dataset comprises daily closing prices retrieved from Yahoo Finance, covering the period from 1 January 2020, to 31 December 2024

**Figure 2:**
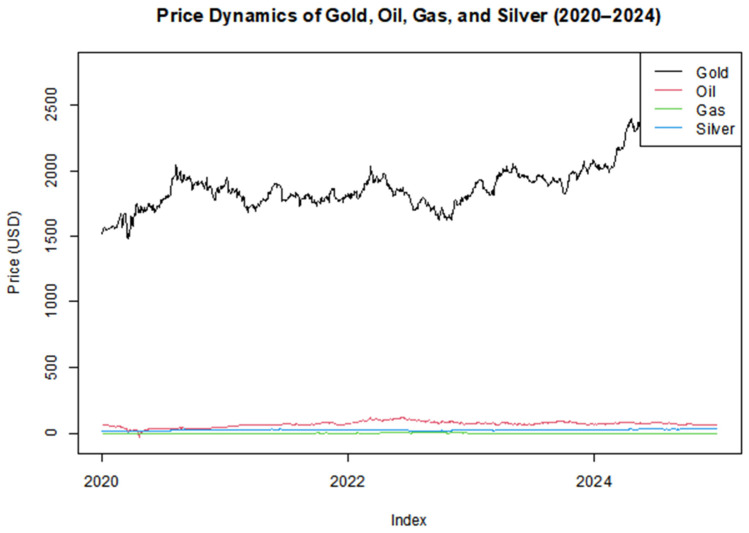
Price Dynamics of Gold, Oil, Gas, and Silver (2020–2024).

Figure 2 illustrates the daily price trajectories of four major commodities—Gold, Oil, Natural Gas, and Silver—over the period from January 2020 to December 2024. Gold, shown in black, exhibits a markedly higher price level compared to the other three assets, reflecting its role as a traditional safe haven, particularly during episodes of financial or geopolitical uncertainty. Its upward trend in late 2023–2024 may be attributed to inflationary concerns, market volatility, or monetary policy shifts.

Oil prices (in red) show considerable fluctuations, peaking around 2022 due to post-pandemic recovery and energy supply constraints, followed by moderate stabilization. Gas (green) and Silver (blue) remain comparatively low in absolute value, but also display noticeable volatility, especially during the 2021–2022 period—possibly linked to disruptions in energy supply chains and global demand pressures.

The visual divergence in scale and behavior among the assets highlights the heterogeneity in their market drivers. While Gold trends upward with relatively less noise, Oil and Gas exhibit higher short-term volatility, consistent with their sensitivity to geopolitical tensions and seasonal demand. Silver shows characteristics somewhere between Gold and industrial commodities, influenced by both investment demand and industrial usage.

Log-returns were computed to eliminate unit root concerns and reflect percentage-based dynamics.

**Figure 3:**
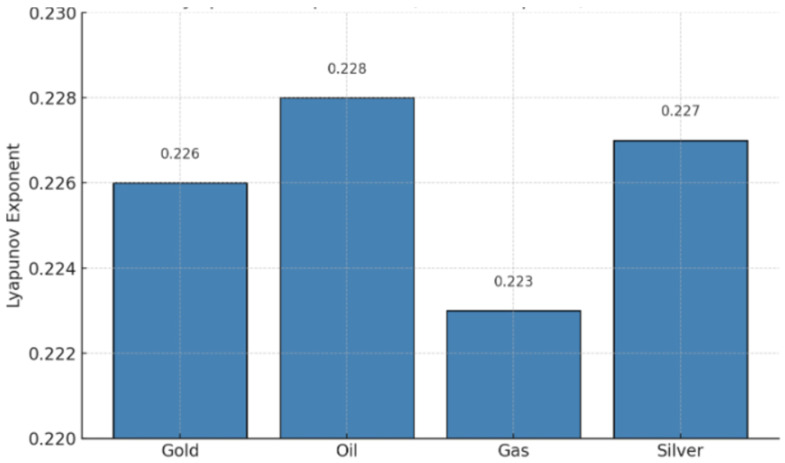
Estimated Lyapunov Exponents (Kantz) For Financial Assets.

The bar plot in Figure 3 visually compares the largest Lyapunov exponents of four financial assets: Gold, Oil, Gas, and Silver. The values are estimated using a Kantz-inspired method on their log-returns. Oil has the highest Lyapunov exponent (0.228), indicating the most pronounced chaotic behavior. Silver and Gold follow closely with values of 0.227 and 0.226, respectively. Gas shows the lowest exponent at 0.223, suggesting relatively less sensitivity to initial conditions, though still within the chaotic regime.

These positive Lyapunov values suggest that all four markets exhibit deterministic chaos, which is consistent with nonlinear and unpredictable dynamics often seen in financial systems.

Table 1 summarizes the complexity and chaotic properties of the four financial assets—Gold, Oil, Natural Gas, and Silver—based on correlation dimension, Shannon entropy, and Rényi entropy. The correlation dimension, estimated using the Grassberger–Procaccia method, provides insight into the fractal structure of the time series. Higher values suggest more complex and chaotic dynamics. Oil and Silver exhibit the highest correlation dimension values (3.35 and 3.01, respectively), indicating rich and possibly more chaotic dynamics in their price movements. These assets likely follow attractors with greater degrees of freedom, implying that their evolution is influenced by multiple interacting components and nonlinear dependencies. In contrast, Gold and Natural Gas show relatively lower correlation dimension values (1.14 and 1.37), suggesting simpler underlying structures or potentially more deterministic behavior. Entropy-based measures further differentiate the informational content of the asset returns. Shannon entropy captures the average uncertainty or disorder within the time series distribution, while Rényi entropy (with order alpha equal to two) emphasizes tail sensitivity and higher-order interactions. Silver and Gold exhibit the highest values for both Shannon and Rényi entropy, implying that these assets are not only more unpredictable but also possess distributions with substantial tail weight or variability. This characteristic may reflect the influence of external shocks, heterogeneous investor behavior, or global macroeconomic uncertainty, all of which can amplify the complexity of price signals in precious metal markets. Conversely, Oil shows relatively low entropy values, particularly for Shannon entropy, indicating that its returns are more concentrated and potentially governed by fewer dominant factors, such as supply–demand balances or geopolitical events. Natural Gas falls somewhere in between, reflecting moderate unpredictability and informational richness.

Table 2 presents the mutual information (MI) matrix, which quantifies the nonlinear dependence between pairs of assets. Unlike linear correlation, MI captures shared information content regardless of whether the relationship is linear or nonlinear. Among the four assets, the strongest dependence is observed between Gold and Silver, with a value of approximately 0.648. This confirms the well-established co-movement between these two precious metals, which often act as investment hedges and safe-haven assets. For other pairs, the MI values are close to zero, and in some cases appear slightly negative (marked with *). As noted under Table 2, these negative values are estimation artifacts caused by finite-sample discretization and should be interpreted as negligible dependence. This suggests that, aside from the Gold–Silver pair, the remaining assets exhibit little to no nonlinear informational linkage over the studied period.

Taken together, these findings highlight the heterogeneity in both the complexity and interdependence of financial assets. Silver and Gold emerge as the most complex in terms of both fractal geometry and entropy content, while Oil and Gas differ in terms of shared information, with Oil displaying complex dynamics but lower unpredictability, and Gas being relatively less connected to other assets. The application of chaos theory and information-theoretic measures such as Lyapunov exponents, entropy, and mutual information offers valuable insight into the nonlinear dynamics of asset behavior and contributes to a richer understanding of systemic risk and portfolio diversification strategies.

**Figure 4:**
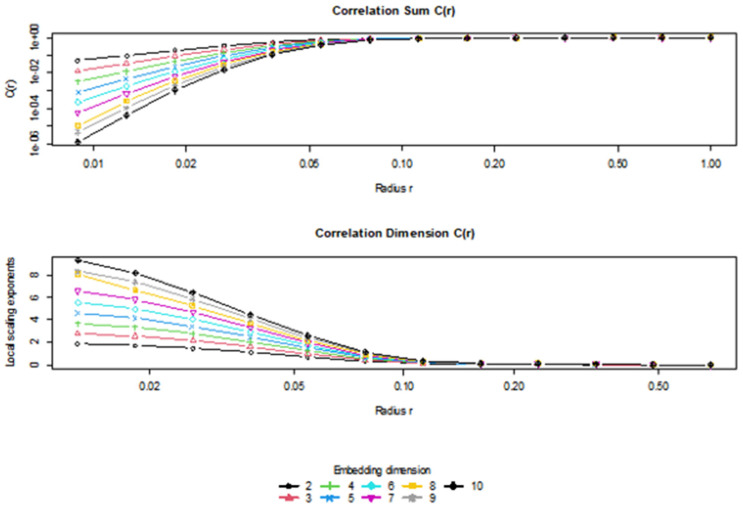
Estimation of the Correlation Dimension Using the Grassberger–Procaccia Method.

Figure 4 presents two diagnostic plots used to estimate the correlation dimension of a time series, based on the Grassberger–Procaccia (G–P) algorithm. The top panel shows the correlation sum C(r) plotted against the radius r on a logarithmic scale for a range of embedding dimensions m = 2 to m = 10. The correlation sum quantifies the likelihood that pairs of points in the reconstructed phase space lie within a given distance r of each other. As expected, C(r) increases monotonically with r, and curves shift upward as the embedding dimension increases. This is consistent with a higher-dimensional attractor structure, as more pairs fall within the radius as m increases.

The bottom panel displays the local slopes of the log–log plot of C(r), which approximate the correlation dimension D2 for different embedding dimensions. A plateau (i.e., a region of the radius r where the local slope becomes relatively constant across dimensions) indicates convergence to the true correlation dimension. In this case, the convergence seems to stabilize between dimensions m = 6 and m = 8, particularly for radii between 0.03 and 0.07, where the local scaling exponents are nearly constant. The estimated correlation dimension can thus be inferred from this stable region, supporting the value reported earlier (e.g., approximately 3–5 depending on the asset).

Figure 4 confirms that the time series exhibits fractal-like scaling behavior, consistent with low-dimensional deterministic chaos. The identification of a stable region suggests a reliable estimate of the attractor’s dimension, justifying the use of nonlinear techniques in analyzing the dynamics of financial time series such as Gold, Oil, Gas, or Silver.

**Figure 5:**
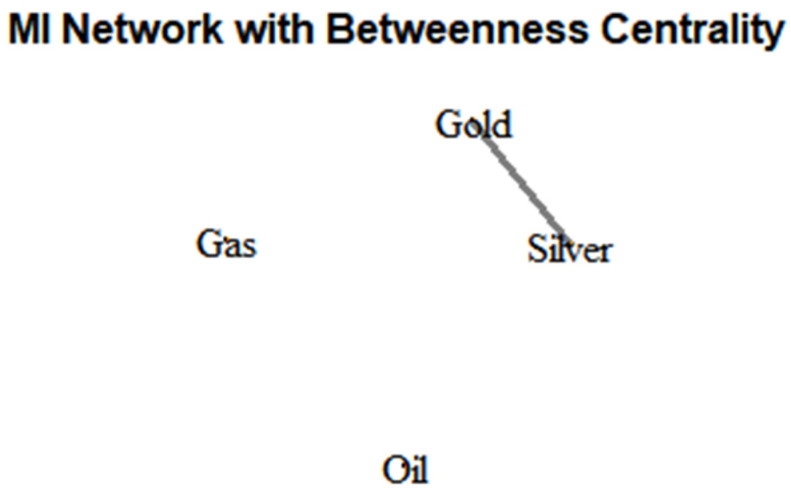
MI network with betweenness centrality.

Figure 5 visualizes the mutual information network constructed from the log-returns of four financial assets—Gold, Silver, Gas, and Oil—based on pairwise MI values exceeding a threshold (here, I > 0.01). The network is undirected and illustrates informational dependencies rather than linear correlations.

Only one edge appears in the network, connecting Gold and Silver, indicating that among the asset pairs, only this pair shares a significant level of mutual information over the observed period (2020–2024). This edge implies a degree of nonlinear dependence between their return dynamics. The presence of this edge is consistent with their economic linkage, as both are precious metals often influenced by similar macroeconomic factors, such as inflation expectations, safe-haven demand, and interest rates.

Betweenness centrality is visualized through node size, but in this sparse graph, where only one connection exists, centrality is trivial—Gold and Silver have equal importance, and Oil and Gas are isolated nodes with no informational connection above the threshold. Their isolation suggests weak or no significant nonlinear dependence with the other assets during the sample period.

In summary, this sparse MI network reveals a dominant informational link between Gold and Silver, while Oil and Gas behave independently in terms of nonlinear information flow. This highlights the unique co-movement structure in the system and supports asset-specific analysis when modeling systemic risk or portfolio optimization using information-theoretic tools.

**Figure 6:**
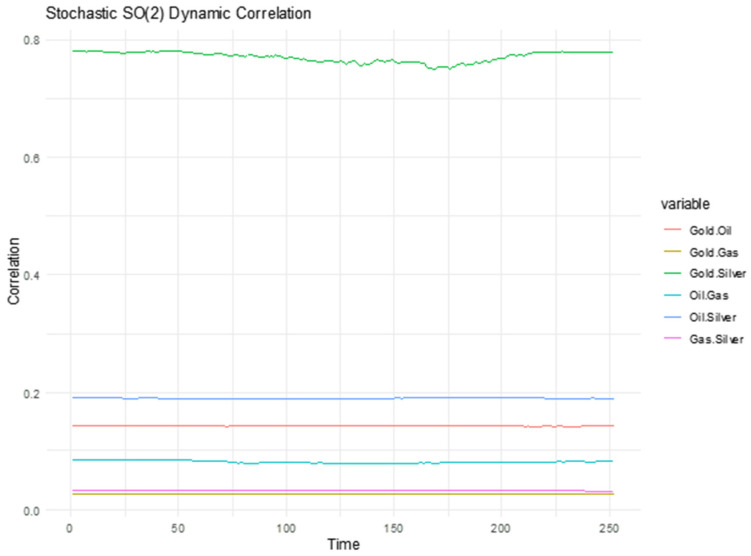
Stochastic SO(2) Dynamic Correlation Between Financial Assets.

Figure 6 displays the dynamic correlations between pairs of financial assets—Gold, Oil, Gas, and Silver—estimated using a stochastic SO(2) Lie group model. The SO(2) framework applies smooth rotational transformations to the correlation matrix, capturing the time-evolving structure of correlations while preserving the geometric constraints of correlation matrices.

From the plot, we observe that the Gold–Silver pair (green line) consistently exhibits the highest correlation, with values fluctuating around 0.77–0.78 throughout the time window. This strong and persistent co-movement is intuitive, as both are precious metals that often respond similarly to macroeconomic factors such as inflation, monetary policy, and safe-haven demand.

The Oil–Gas correlation (blue line) ranks next, maintaining a moderate level around 0.18–0.19, reflecting their shared role in the energy sector and exposure to similar supply-demand shocks, albeit with some differentiation due to distinct market structures and uses (e.g., transport fuel vs. heating/power generation).

All other pairs—Gold–Oil, Gold–Gas, Oil–Silver, and Gas–Silver—display low but stable correlations, generally below 0.15, suggesting weak or limited dynamic co-movement. These relationships are likely influenced by fundamentally different demand drivers, sectoral roles, and risk profiles.

Overall, the SO(2) model captures a stable but heterogeneous correlation structure, emphasizing that only a few asset pairs—especially Gold and Silver—exhibit strong dynamic coupling. This finding supports the idea that while certain commodities are closely tied, others behave more independently, which can be leveraged for portfolio diversification and risk management purposes

**Figure 7:**
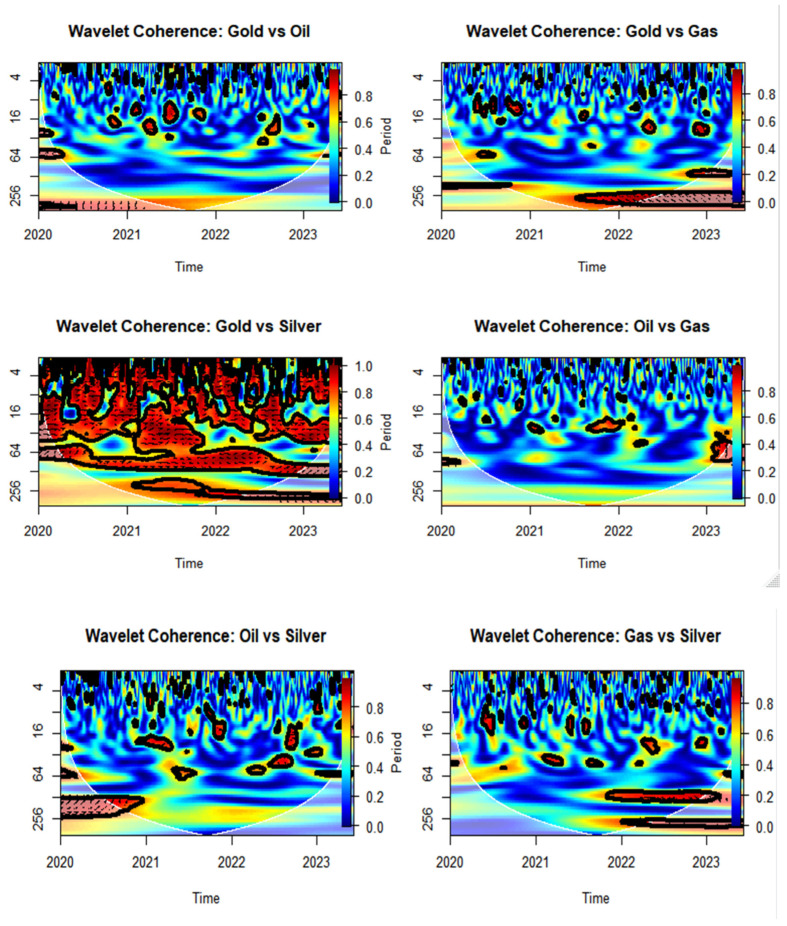
Time–Frequency Dependency via Wavelet Coherence.

The wavelet coherence plots in Figure 7 analyze the dynamic co-movement between log-returns of four assets—Gold, Oil, Gas, and Silver—over the period from 2020 to 2024, across different time scales. Each plot reveals not only how the correlation between assets evolves over time, but also at what frequencies (or periodicities) the assets exhibit significant coherence. Warm colors (yellow/red) denote strong coherence, while cooler colors (blue) indicate weaker or no coherence. Black contour lines show statistically significant regions, and arrows provide information about the phase relationship between the series.

Among all the pairs, the strongest and most persistent relationship is observed between Gold and Silver. This pair displays extensive regions of high coherence, particularly in the mid- and low-frequency bands, suggesting a stable and significant long-term and medium-term co-movement. This is consistent with market expectations, as both are precious metals often influenced by similar economic and geopolitical factors, such as inflation expectations, monetary policy, and safe-haven demand.

The Oil and Gas pair also shows areas of moderate to high coherence, particularly around late 2020 and early 2022, at lower frequencies (longer time scales). This pattern reflects the structural and economic link between these two energy commodities, which are often affected by common supply and demand shocks, geopolitical tensions, and seasonality in energy consumption.

Other pairs, such as Gold and Oil, Gold and Gas, Oil and Silver, and Gas and Silver, show weaker and more intermittent coherence. These relationships are mostly limited to isolated periods and are significant only at lower frequencies (long-term scales). For instance, Gold and Oil display a brief period of moderate coherence during mid-2020 and again in mid-2022, likely reflecting broad market reactions to global crises like COVID-19 or energy shocks. Meanwhile, Oil and Silver and Gas and Silver exhibit only scattered coherence zones, suggesting that their co-movement is largely episodic and not structurally linked.

Overall, the wavelet coherence analysis highlights that while some asset pairs such as Gold–Silver and Oil–Gas exhibit strong and consistent co-movement patterns, most other combinations show only limited or context-specific coherence. This suggests that portfolio diversification using these assets may benefit from combining those with low coherence, especially at the desired investment horizon.

## 5. Conclusions

This study presents a multidimensional analysis of chaotic behavior, nonlinear dependence, and time–frequency co-movement among Gold, Oil, Gas, and Silver markets. The results provide strong evidence of deterministic chaos as indicated by positive Lyapunov exponents and high correlation dimensions, especially in the cases of Oil and Silver. Entropy analysis shows notable unpredictability in asset returns, with Silver and Gold exhibiting the highest informational complexity.

The mutual information framework reveals significant nonlinear connections, particularly between Gold and Silver, while the stochastic SO(2) Lie group rotation model captures dynamic and geometry-preserving correlations that vary meaningfully over time. Wavelet coherence plots further confirm that co-movement among certain asset pairs, such as Gold–Silver and Oil–Gas, is both time-dependent and frequency-specific, underscoring the multifaceted nature of financial interdependencies.

From a practical standpoint, these findings have direct implications for portfolio construction, hedging strategies, and systemic risk assessment. Investors and policymakers should consider the dynamic and nonlinear structure of inter-asset relationships, especially under crisis conditions, where traditional correlation-based tools may fail to detect hidden linkages.

Future research could extend this framework to include higher-order Lie groups (e.g., SO(3), SU(n)) and investigate network-based representations of mutual information across broader asset classes, including equities, cryptocurrencies, and ESG investments. Incorporating machine learning techniques to model the evolution of entropy or dynamic correlations, or combining nonlinear diagnostics with regime-switching models, could offer additional predictive power. Furthermore, investigating the causal structure using transfer entropy or directed information could deepen our understanding of lead–lag dynamics in interconnected markets.

## Figures and Tables

**Table 1:** Chaos metrics table.

Asset	CorrDim	Shannon	Renyi
Gold	1.135925	2.0715518	1.809249
Oil	3.350946	0.9697773	0.814083
Gas	1.365901	1.3659301	1.159213
Silver	3.005377	2.0957338	1.819953

**Table 2:** Mutual matrix information. Negative values marked with * are estimation artifacts and indicate negligible dependence.

	Gold	Oil	Gas	Silver
Gold		−0.199 *	−0.2083 *	0.648
Oil	−0.199 *		−0.671 *	−0.2245 *
Gas	−0.2083 *	−0.671 *		−0.233 *
Silver	0.648	−0.2245 *	−0.233 *	

* Note: By definition, mutual information (MI) is non-negative. The negative values (marked with *) are artifacts of finite-sample discretization and estimation noise. They should be interpreted as values close to zero, i.e., negligible dependence.

## Data Availability

Data is public from Yahoo Finance, available upon request.

## Appendix A

### Appendix A.1. Lyapunov Exponent—Technical Details

A system can have multiple Lyapunov exponents (one for each dimension of the state space), but the largest one—called the largest Lyapunov exponent (LLE)—is typically used as a benchmark for chaos detection.

The value of the Lyapunov exponent λ provides insight into the nature of the dynamical system. Specifically, λ > 0 indicates the presence of chaotic behavior characterized by sensitive dependence on initial conditions, a hallmark of deterministic chaos. In contrast, λ = 0 reflects marginally stable dynamics such as quasiperiodic motion or bifurcating systems, while λ < 0 suggests convergence toward stable fixed points or limit cycles. In financial time series analysis, a positive Lyapunov exponent has often been interpreted as evidence of deterministic yet unpredictable dynamics, particularly in domains such as asset pricing, interest rate modeling, and exchange rate volatility (Peters, 1994 [19]; Scheinkman & LeBaron, 1989 [20]).

In practice, the Lyapunov exponent is estimated from empirical time series data using phase-space reconstruction techniques. One widely used approach is the Kantz algorithm, which reconstructs the system’s attractor via time-delay embedding and measures the divergence of neighboring points over time (Kantz, 1994 [29]). The estimation requires a selection of parameters such as embedding dimension m, time delay τ, and neighborhood radius ϵ. These are often chosen using mutual information (for τ) and false nearest neighbors (for m) to ensure optimal embedding (Fraser & Swinney, 1986 [30]; Kennel et al., 1992 [31]).

The Kantz method (Kantz, 1994 [29]) is employed to estimate the largest Lyapunov exponent from a scalar time series via phase space reconstruction using Takens’ embedding theorem (Takens, 1981 [32]). For a scalar time series $\left\{x_{t}\right\}$, we reconstruct the phase space using delay vectors:

(A1) $$x_{t}=\left(x_{t},x_{t+\tau },x_{t+2\tau },\ldots ,{\;x}_{t+\left(m-1\right)\tau }\right)$$

where m is the embedding dimension and τ is the time delay. The LLE is defined as the average exponential rate of separation of nearby trajectories:

(A2) $$\lambda =\underset{t\to \infty }{\lim}\frac{1}{t}\langle log\frac{d_{i}\left(t\right)}{d_{i}\left(0\right)}\rangle$$

In this formulation, $d_{i}\left(t\right)\;$denotes the distance between two initially close trajectories $x_{i}$ and $x_{j}$ in the reconstructed phase space after evolving for time t. These distances are typically computed using the Euclidean norm. The function $S\left(t\right)=\langle \log d_{i}\left(t\right)\rangle =\frac{1}{N}\Sigma_{i=1}^{N}\log d_{i}\left(t\right)$ represents the average logarithmic divergence of all such pairs, where the angle brackets ⟨⋅⟩ indicate averaging over all valid trajectory pairs. The largest Lyapunov exponent λ is estimated by identifying the linear portion of the S(t) versus t curve and computing its slope, which reflects the average exponential rate at which nearby trajectories in phase space diverge. This procedure quantifies how quickly nearby trajectories separate, which is a hallmark of chaotic dynamics.

A positive LLE implies the presence of deterministic chaos, while values near zero or negative suggest non-chaotic or stable dynamics.

We apply this method using the maxLyapunov() function from the nonlinearTseries package in R, with parameters: embedding dimension m = 2, delay τ = 1, and radius ϵ = 0.05, consistent with the guidelines in Kantz (1994) [29] and Kantz & Schreiber (2003) [33].

### Appendix A.2. Correlation Dimension (Grassberger–Procaccia Method)–Technical Details

This method is widely used in empirical studies to assess the presence of deterministic chaos and to distinguish between regular, chaotic, and stochastic dynamics in time series data.

To empirically estimate the correlation dimension D_2_ of each asset’s log-return time series, we employed the Grassberger–Procaccia algorithm implemented via the nonlinearTseries package in R. Following the reconstruction of the phase space using delay embedding, the correlation sum C(r) was computed over a range of radii r, and the slope of the log–log plot log C(r) versus log r was used to estimate the correlation dimension.

The estimation procedure involved the following steps in R:

- Phase Space Reconstruction

The time series for each asset was embedded in dimensions m = 2 to m = 10, with a fixed time delay τ = 1. This was performed using the function corrDim() from the nonlinearTseries package in R.

- Correlation Dimension Estimation

After computing the correlation sums C(r), the dimension D_2_ was estimated using linear regression over the identified scaling region, using the function estimate() and regression range ϵ ∈ [0.01, 0.1].

- Interpretation

The resulting D_2_ values were interpreted as indicators of system complexity. Values significantly greater than 2 suggest the presence of deterministic chaos or high-dimensional fractal structures, while low values indicate regular or stochastic dynamics.

### Appendix A.3. Mutual Information–Technical Details

In practice, we estimated the entropy terms by discretizing the time series using histogram binning, calculating empirical probabilities, and applying the standard Shannon entropy formula. MI was computed based on Shannon entropy (Shannon, 1948 [22]) and further formalized in modern treatments of information theory (Cover & Thomas, 2006 [23]).

The MI implementation in R allowed us to construct mutual information networks between assets, visualized by igraph in R, and further analyzed using centrality measures. By capturing nonlinear dependencies, mutual information complements traditional correlation analysis and enhances understanding of the dynamic interconnectivity in financial systems.

### Appendix A.4. Dynamic Correlations via Lie Group SO(2) Rotations—Technical Details

The model begins with a fixed initial covariance matrix $C_{0}$ (typically computed from a short time window of observed returns). We then evolve this matrix via time-dependent rotations:

(A3) $$C_{t}=A{\left(\theta_{t}\right)}^{T}C_{0}A\left(\theta_{t}\right)$$

where $A\left(\theta_{t}\right)\in SO\left(2\right)$ is a rotation matrix defined as:

(A4) $$A\left(\theta \right)=\left(\begin{array}{cc} \cos\theta & -\sin\theta \\ \sin\theta & \cos\theta \end{array}\right)$$

The angular parameter $\theta_{t}$ evolves stochastically over time according to a Brownian motion with drift:

(A5) $$\theta_{t}=\mu \mathrm{dt}+{\sigma \mathrm{dW}}_{t}$$

In Equation (A5), μ represents the drift term, σ denotes the volatility, and $dW_{t}$ is a Wiener process. This stochastic evolution allows the correlation matrix to adapt over time, capturing the dynamic nature of financial markets.

To obtain a normalized and interpretable measure of dynamic dependence, we compute the time-varying correlation coefficient from the rotated covariance matrix $C_{t}$ using the following expression:

(A6) $$\rho_{12}\left(t\right)=\frac{C_{t}\left[1,2\right]}{\sqrt{C_{t}\left[1,1\right]\cdot C_{t}\left[2,2\right]}}$$

Formula (A6) extracts the correlation between two assets at time t, ensuring the result is bounded in the interval [−1, 1], as required for meaningful correlation interpretation. The denominator normalizes the covariance by the geometric mean of the two variances, similar to the standard Pearson correlation, but here dynamically adjusted using the rotated covariance matrix $C_{t}$.

By adopting this approach, we aim to model the dynamic correlations between assets such as gold, oil, natural gas, and silver, providing insights into their interdependencies and how they evolve in response to market conditions.

The SO(2) rotation framework offers key advantages for modeling time-varying correlations. First, it preserves the positive-definiteness of the covariance matrix, ensuring the resulting correlation matrices are valid throughout the simulation. Second, it is reversible and grounded in group theory, providing a geometric interpretation of correlation dynamics. Third, the model introduces stochastic yet structure-preserving evolution, overcoming limitations of standard methods like rolling correlations or random walks.

It should be noted that the SO(2) rotations applied here operate at the pairwise level, ensuring valid 2 × 2 correlation blocks but not guaranteeing the existence of a single consistent 4 × 4 correlation matrix across all assets. Our network representation should therefore be interpreted as a visualization of bilateral dynamic dependencies. Extensions to higher-order Lie groups (e.g., SO(3), SO(4)) can provide such global consistency, as proved by Bildirici et al. (2022) [34] in their application of SO(3) to interest rate dynamics.

### Appendix A.5. Time–Frequency Dependency via Wavelet Coherence—Technical Details

The continuous wavelet transform (CWT) of a time series x(t) using a mother wavelet ψ is defined as:

(A7) $$W_{x}\left(u,s\right)=\frac{1}{\surd s}\int_{-\infty }^{\infty }x\left(t\right)\psi^{*}\left(\frac{t-u}{s}\right)\mathrm{dt}$$

where u is the time position, s is the scale (inversely related to frequency), and ψ* denotes the complex conjugate of the mother wavelet function. We use the Morlet wavelet, a complex sine modulated by a Gaussian envelope, due to its optimal time–frequency localization properties.

For two time series x(t) and y(t), the wavelet coherence is defined as:

(A8) $$R^{2}\left(u,s\right)=\frac{{\left|S\left(s^{-1}W_{\mathrm{xy}}\left(u,s\right)\right)\right|}^{2}}{S\left(s^{-1}{\lceil W_{x}\left(u,s\right)\rceil }^{2}\right)\cdot S\left(s^{-1}{\lceil W_{y}\left(u,s\right)\rceil }^{2}\right)}$$

where $W_{\mathrm{xu}}\left(u,s\right)=W_{x}\left(u,s\right)W_{n}^{*}\left(u,s\right)$ is the cross-wavelet transform and S is a smoothing operator in both time and scale. The resulting coherence values $R^{2}\left(u,s\right)\in \left[0,1\right]$ describe the local correlation strength. High coherence values indicate strong time- and scale-specific dependence between the series (Aguiar-Conraria & Soares, 2011 [10]).

In addition to the magnitude of coherence, the phase difference between the time series is also examined. This provides insight into the direction of the relationship—whether the series move together (in-phase), in opposite directions (anti-phase), or whether one leads the other. These relationships are represented graphically through arrows in the wavelet coherence plots, enabling the identification of lead–lag structures in different periods and frequencies (Rua & Nunes, 2009) [35].

To assess statistical significance, we use Monte Carlo simulation techniques with red noise as the null hypothesis. This ensures that the observed coherence patterns are not merely due to random fluctuations, but reflect meaningful dynamic associations. Statistically significant regions are highlighted with contour lines in the coherence plots, following the approach proposed by Torrence and Webster (1999) [36].

The empirical implementation was carried out using the biwavelet package in R. First, the asset price series were transformed into stationary log-returns. Then, the continuous wavelet transforms and cross-wavelet spectra were computed for all asset pairs. Finally, squared wavelet coherence and phase difference estimates were obtained, and significance testing was applied using Monte Carlo resampling with 1000 surrogate data series.
